# A Sheet-like Carbon Matrix Hosted Sulfur as Cathode for High-performance Lithium-Sulfur Batteries

**DOI:** 10.1038/srep20445

**Published:** 2016-02-04

**Authors:** Songtao Lu, Yan Chen, Jia Zhou, Zhida Wang, Xiaohong Wu, Jian Gu, Xiaoping Zhang, Aimin Pang, Zilong Jiao, Lixiang Jiang

**Affiliations:** 1 Department of Chemistry, Harbin Institute of Technology, Harbin, Heilongjiang 150001, PR China; 2 Hubei Institute of Aerospace Chemotechnology, Hubei 441003, PR China; 3 Science and Technology on Reliability and Environmental Engineering Laboratory, Beijing Institute of Satellite Environment Engineering, Beijing, 100094, PR China

## Abstract

Lithium-sulfur (Li-S) batteries are a promising candidate of next generation energy storage systems owing to its high theoretical capacity and energy density. However, to date, its commercial application was hindered by the inherent problems of sulfur cathode. Additionally, with the rapid decline of non-renewable resources and active appeal of green chemistry, the intensive research of new electrode materials was conducted worldwide. We have obtained a sheet-like carbon material (shaddock peel carbon sheets SPCS) from organic waste shaddock peel, which can be used as the conductive carbon matrix for sulfur-based cathodes. Furthermore, the raw materials are low-cost, truly green and recyclable. As a result, the sulfur cathode made with SPCS (SPCS-S), can deliver a high reversible capacity of 722.5 mAh g^−1^ at 0.2 C after 100 cycles with capacity recuperability of ~90%, demonstrating that the SPCS-S hybrid is of great potential as the cathode for rechargeable Li-S batteries. The high electrochemical performance of SPCS-S hybrid could be attributed to the sheet-like carbon network with large surface area and high conductivity of the SPCS, in which the carbon sheets enable the uniform distribution of sulfur, better ability to trap the soluble polysulfides and accommodate volume expansion/shrinkage of sulfur during repeated charge/discharge cycles.

With the notable scaled-up demand of rechargeable batteries with high energy and power density for various consumer electronic applications, lithium-sulfur (Li-S) batteries have attracted enthusiastic interest as powerful energy storage systems owing to their overwhelmingly high theoretical gravimetric capacity (1672 mAh g^−1^) and energy density (2580 Wh kg^−1^)^1^,^2^. Furthermore, sulfur is naturally abundant, low-cost, and environmentally friendly, making Li-S batteries promising candidates of next generation battery systems^3^,^4^. Unfortunately, several challenges must be addressed prior to its commercial application^5^,^6^,^7^,^8^. The first is the intrinsic insulating nature of sulfur and lithium sulfides, which hinders the complete conversion of sulfur to lithium sulfides once lithium sulfides layer (Li_2_S and Li_2_S_2_) covered on the cathode surface, and leads to a low utilization of sulfur and limites specific capacity of cathodes^7^. Moreover, the dissolution and so-called shuttle effect of polysulfides cause the loss of sulfur active materials, the passivation of lithium anode and the increasing impedance of cells as these soluble sulfur species reduce to lithium sulfides on the surface of lithium anode^1^. Finally, the severe volume shrinkage/expansion of sulfur species during charge/discharge process results in the gradual alteration of morphology and integrity of cathode^2^.

Over the past few decades, the carbon (such as carbon black^9^,^10^, graphene^11^,^12^, carbon nanotubes^13^,^14^,^15^, carbon nanofibers^16^,^17^, and porous carbon spheres^18^,^19^,^20^) modified sulfur-based cathodes has demonstrated a significantly-improved cyclability and capacity. Most recently, apart from conventional carbon materials, biomass carbon has shown its prospects in electrochemical energy systems due to their abundance, inexpensive cost and truly environmental benignity^21^,^22^,^23^,^24^,^25^,^26^,^27^. For instance, the peanut shells are a green and economical waste, with a high yield over 6 million tons per year globally. Ding *et al.* used the carbon cmaterials derived entirely from peanut shells, as both anode and cathode for sodium ion capacitor, and the electrodes delivered a promising electrochemical performance^28^. In addition, Zhang *et al.* applied porous carbon materials, prepared from silk cocoon, to encapsulate sulfur that used as cathode for Li-S batteries^29^. In their work, the carbon-sulfur composites exhibited a specific capacity of 804 mAh g^−1^ with a Coulombic efficiency of 92% after 80 cycles at a rate of 0.5 C. Shaddock is a kind of popular fruits, with the global production over 8 million tons in 2012 alone. Shaddock peel (SP) is composed of cellulose, pectin, low molecular weight hydro-carbons, etc., and usually considered as a waste. Given the huge amount of the yield of shaddock per year, it becomes extremely important to make use of SP from not only economical perspective but environmental perspective. So far, due to its active groups (such as hydroxyl and carboxyl groups), as well as its porous and spongy structure, SP has been not only utilized as an effective adsorbent for removing methylene blue from aqueous solution^30^ and a toxic radioactive heavy metal (Uranium VI)^31^, but also served as electrode material for batteries^32^,^33^.

Herein, we employ hydrothermal treatment and carbonization (see the experimental section for details) to take full advantage of the unique structure of SP and ultimately achieve a sheet-like carbon material with 3D interconnected framework composed of transparent and crumpled carbon sheets, namely Shaddock Peel Carbon Sheets SPCS. The SPCS-sulfur (SPCS-S) hybrid obtained by the method of melting diffusion, with even distribution of sulfur, can deliver an excellent electrochemical performance. For instance, the SPCS-S cathode with 62.0 wt% sulfur can deliver a high reversible capacity of 722.5 mAh g^−1^ at 0.2 C after 100 cycles, a capacity recuperability of ~90% as the current returned to 0.2 C and a high Coulombic efficiency of ~99.5%.

## Results and Discussion

**Figure 1:**
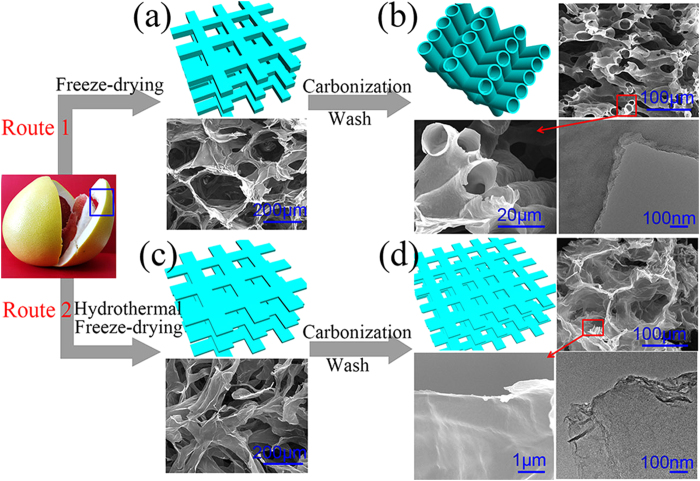
Materials synthesis process employed for SPCT (Route 1) and SPCS (Route 2). Models, SEM and TEM images: (**a**) SP after freeze-drying; (**b**) SPCT; (**c**) SP after hydrothermal treatment and freeze-drying; (**d**) SPCS.

**Figure 2:**
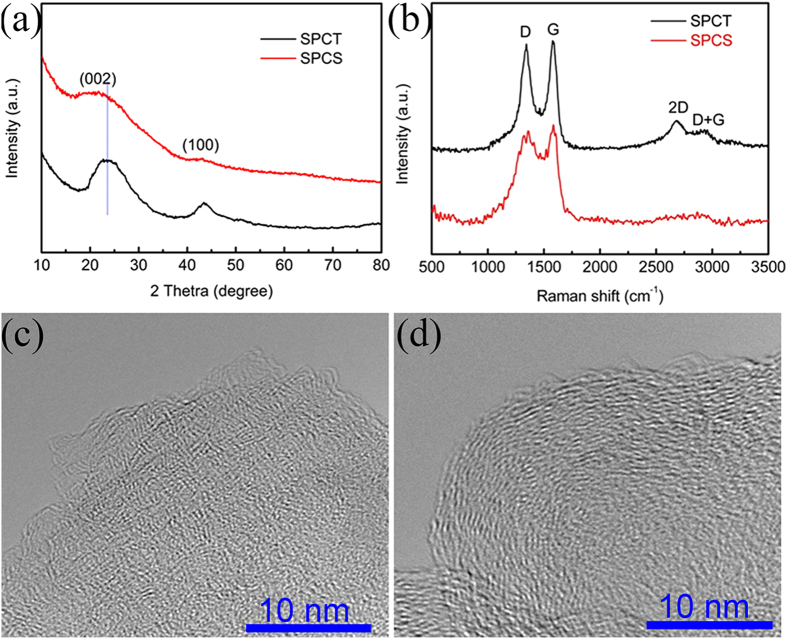
Carbon structure characterization (**a**) XRD patterns, (**b**) Raman spectrum, HRTEM images of (**c**) SPCS and (**d**) SPCT.

Two fundamentally different structures of carbon materials were achieved via tailoring the synthesis process from the same precursor SP. Figure 1 illustrates each carbon material synthesis process employed for sulfur electrode, as well as the relevant scanning electron microscopy (SEM) images. The SP was firstly freeze-dried and then carbonized at argon and hydrogen atmosphere (Route 1 in Fig. 1). For comparison, another carbon material was prepared by hydrothermal treatment, followed by freeze-dying and carbonization at same condition (Route 2 in Fig. 1). Both products were washed with dilute hydrochloric acid (HCl) to remove potential impurities that may be present in plant-based precursors and then rinsed with abundant DI water. From Fig. 1a, the freeze-dried SP shows a porous and spongy structure. After carbonization in Route 1, the products consisted of interconnected carbon tubes with a diameter of ~20 μm are shown in Fig. 1b, namely Shaddock Peel Carbon Tubes (SPCT). The tubes of SPCT are not only in favor of infusing of sulfur and infiltrating of electrolyte, but also suitable for accommodating the volume changes of sulfur species during charge/discharge process. In Route 2, the SP was pretreated by hydrothermal treatment ahead of freeze-drying, in which the SP was exfoliated into sheets as shown in Fig. 1c. After carbonization, as shown in the high magnification SEM image and TEM image (Fig. 1d), the products consisted of transparent and crumpled carbon sheets are detected, which indicates its few layers of carbon atom and the two-dimensional structure, namely Shaddock Peel Carbon Sheets (SPCS). The 3D interconnected porous network of SPCS suggests a high specific surface area, a large sulfur loading, and an achievable permeation of electrolyte. For SPCS (Route 2), the exfoliated sheet-like material was obtained after hydrothermal treatment, which was demonstrated directly by the TEM characterization of SPCT and SPCS in Fig. 1b,d, and further demonstrated by the surface chemical constituents and specific surface area analyses (the higher oxygen content and specific surface area after hydrothermal treatment as shown in Table 1) and the interlayer spacing of SPCT and SPCS determined by the X-ray powder diffraction (XRD) results (Fig. 2a, discussed later in the text).

The surface elemental analysis of samples was carried out by using X-ray photoelectron spectroscopy (XPS) and energy-dispersive X-ray spectroscopy (EDS) with SEM, listed in Table 1 and Supplementary Information Table S1. Based on the XPS and EDS analysis, the content of oxygen increased after hydrothermal treatment, confirming the increasing degree of oxidation of SP. For SPCT and SPCS, the C/O ratio was clearly enhanced after carbonization, and the potential impurities (e.g. Mg, Ca, K, P) were not detected, which may be below the detection limits of XPS and EDS analysis. In addition, a small quantity of nitrogen was detected, which would improve adsorption ability, surface polarity, and electron conductivity of carbon materials^34^. Moreover, it has been reported that N-doping could enhance the reduction of sulfur, and provide high discharge potential and initial discharge capacity^35^,^36^. Additionally, the higher K content in SP with hydrothermal treatment was observed owing to the absorption of potassium hydroxide (KOH) on the surface of SP during hydrothermal treatment, which implied that SPCS possessed a higher specific surface area after carbonization because of the chemical activation of KOH to carbon^37^.

The surface area and pore volume of samples were investigated by nitrogen adsorption-desorption test, and the results are collected in Table 1. A higher specific surface area was obtained for SP after hydrothermal treatment than SP without hydrothermal treatment, demonstrating the formation of the exfoliated SP. The highest specific surface area (937 m^2^ g^−1^) and total pore volume (0.8 cm^3^ g^−1^), both of which were desirable for increasing the total ion adsorption and accelerating the electrolyte diffusion, were achieved in SPCS, ascribing to the KOH activation during hydrothermal treatment and carbonization. During carbonization process, KOH etches carbon atoms from ordered carbon structure, leading to an increasing extent of amorphous structure for SPCS^38^.

The structural differences between SPCT and SPCS were inspected by XRD patterns and Raman spectroscopy (Fig. 2). The XRD patterns of SPCT and SPCS (Fig. 2a), show two broad diffraction peaks that are indexed as (002) and (100) of the pretended graphitic domains^28^, however, barely consequential in SPCS, revealing its high amorphous structure. On the other hand, the average carbon interlayer spacing (Table 1) can be calculated from the centre position of (002) peak, the interlayer spacing for SPCS is significantly larger than that of SPCT, demonstrating the exfoliation of SP by hydrothermal treatment and carbonization. The structure of SPCT and SPCS was further monitored by Raman spectroscopy. As shown in Fig. 2b, both samples exhibit broad disorder-induced D-band (~1346 cm^−1^) and in-plane vibration of sp^2^-hybridized carbon G-band (~1582 cm^−1^). The intensity of G-band/D-band ratio, obtained by fitting the spectra, is employed to index the degree of graphitization, as shown in Supplementary Information Figure S1^39^. For SPCT, the value is 1.02, higher than that of SPCS (0.44, Table 1), implying a larger extent of amorphous structure of SPCS^39^. Moreover, the obvious 2D band and D+G band at ~2600–3000 cm^−1^ show a more ordered graphitic structure for SPCT^40^. The XRD and Raman results were confirmed once again by the high resolution transmission electron microscopy (HRTEM) of SPCS (Fig. 2c) and SPCT (Fig. 2d), demonstrating the low ordered and high amorphous structure for SPCS and the high degree of graphitization for SPCT. The SPCS displays an excellent electrical conductivity of 107.2 S cm^−1^ (Table 1), collected by Hall measurement. Nevertheless, the conductivity of SPCT was not able to be obtained account of the failure of pressing SPCT into dense block in order to satisfy requirement of test. The SPCS, SPCT and carbon black electrodes were prepared and assembled cells as same as the sulfur electrodes (see Experimental Section for details), for carrying out EIS test. The Nyquist plot curves were shown in Supplementary Information Figure S2. All electrodes exhibit the insertion of the Nyquist plot with the x-axis in the high-frequency, one compressed semicircle in the middle-frequency and a sloping straight line in the low-frequency. The semicircle in the middle-frequency can be attributed to the charge-trnasfer resistance (*R*_*ct*_). The *R*_*ct*_ value of the cell assembled using the SPCT electrode is lower than that of the cell using carbon black electrodes.

**Figure 3:**
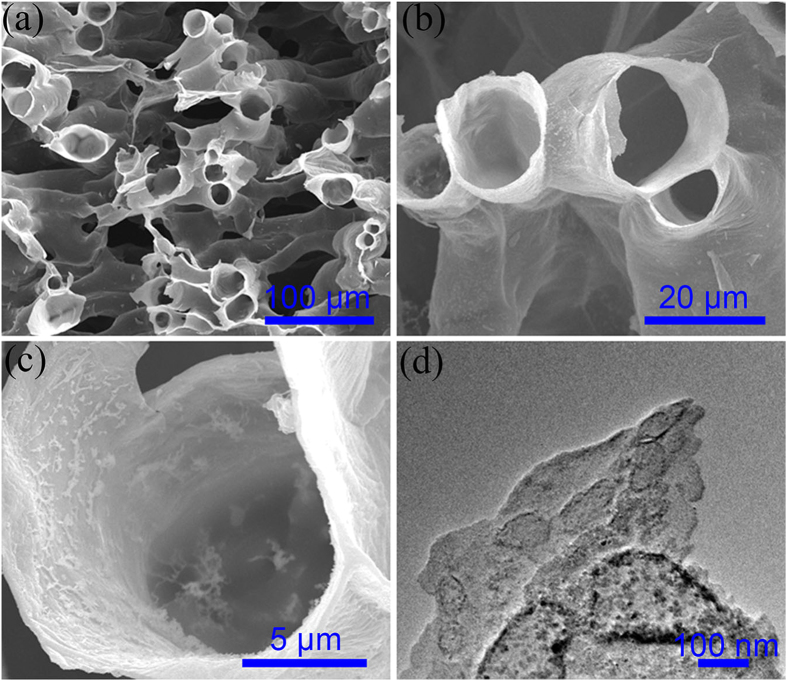
Morphology characterization (**a**,**b**,**c**) SEM images and (**d**) TEM images of SPCT-S hybrid.

**Figure 4:**
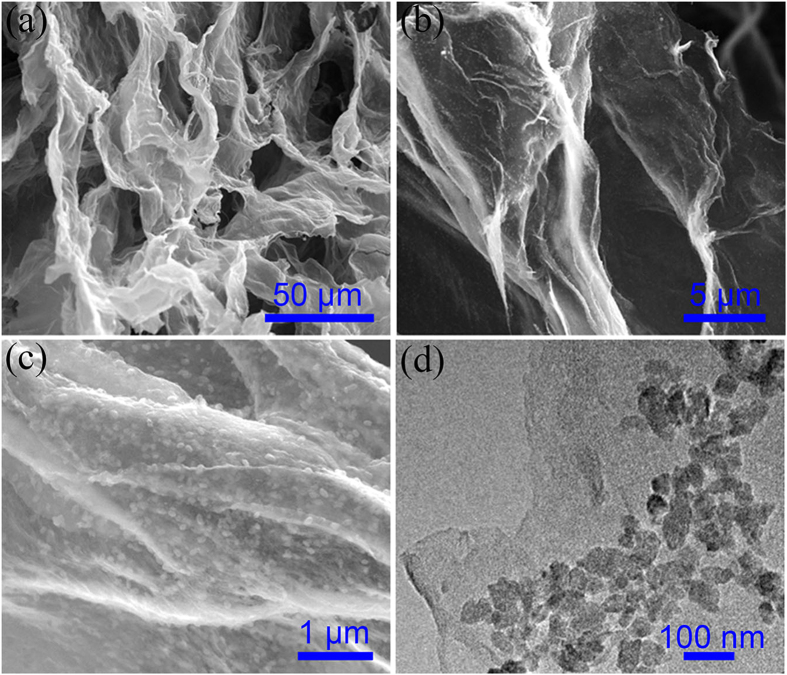
Morphology characterization (**a**,**b**,**c**) SEM images and (**d**) TEM images of SPCS-S hybrid.

The SPCT-sulfur (SPCT-S) and SPCS-sulfur (SPCS-S) hybrids were prepared by a melting diffusion method, at 155 °C for 10 h under vacuum condition. The sulfur content of SPCT-S and SPCS-S was 63.2 wt% and 62.0 wt%, respectively, measured by thermogravimetric analysis (TGA, Supplementary Information Figure S3). For the SPCS-S and SPCT-S, XRD studies (Supplementary Information Figure S4) depicted that no obvious change of characteristic peak of carbon matrix was found, and the sulfur showed sharp and strong peaks, indicated its well-defined crystal structure. Figure 3 shows the SEM images and TEM image of SPCT-S hybrid. The well retained structure of SPCT after incorporating with sulfur is clearly observed from the SEM images (Fig. 3a,b). From the high magnification SEM image (Fig. 3c) and TEM image (Fig. 3d), the mild aggregation of sulfur particles in the channel can be clearly observed, which was further confirmed by the elemental mapping of SPCT-S (Supplementary Information Figure S5) and the high magnification SEM images of SPCT-S from different regions (Supplementary Information Figure S6). Figure 4 displays the SEM images and TEM image of SPCS-S hybrid. It is obvious that the 3D interconnected network structure of SPCS is maintained along with the incorporation of sulfur. The high-magnification SEM image in Fig. 4b,c, illustrates the homogeneous distribution of sulfur particles in SPCS-S hybrid, further confirmed by TEM characterization. The elemental mapping of SPCS-S was shown in Supplementary Information Figure S7. As shown in Fig. 4d, a number of sulfur particles with homogeneous size of ~50 nm are evenly anchored on the surface of carbon sheets in SPCS-S hybrid, which results in an increasing interface of carbon substrate and sulfur, and reveals the increasingly electrochemical reaction sites and indicates the improved capacity and rate property of SPCS-S hybrid as well^41^.

**Figure 5:**
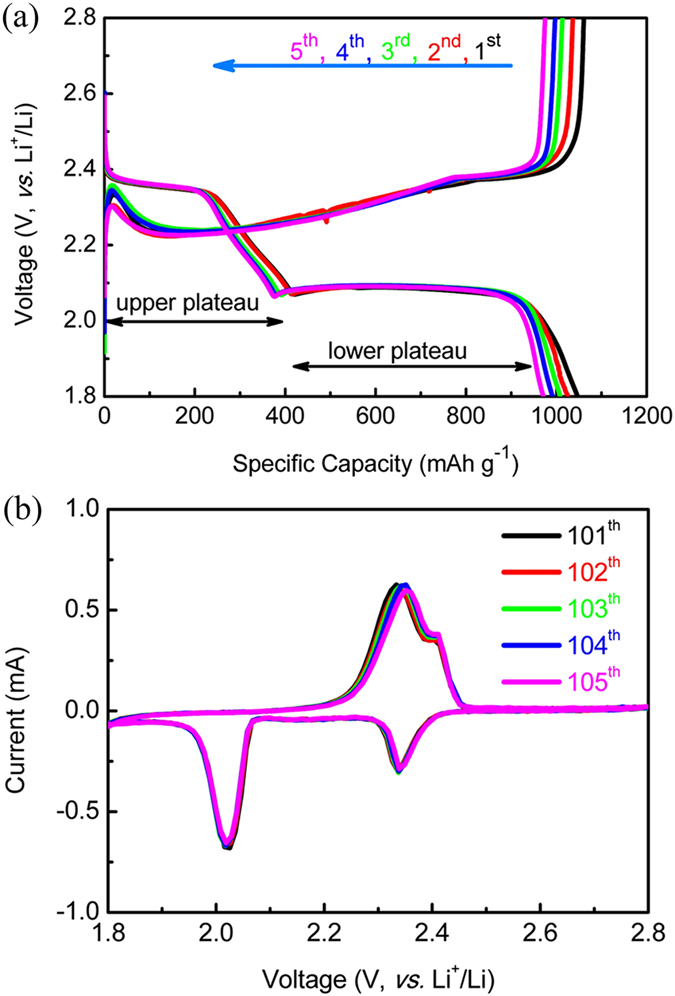
Electrochemical properties (**a**) the galvanostatic charge/discharge curves and (**b**) the cyclic voltammogram curves of SPCS-S hybrid.

In order to investigate the electrochemical properties of the as-prepared SPCT-S and SPCS-S hybrids, coin cells (tape2032) were assembled and tested with a small piece of lithium foil used as the counter electrodes, while a solution of 1 M lithium bis(trifluoromethane) sulfonamide in 1,2-dimethoxymethane: 1,3-dioxolane (1:1, v-v) with 0.1 M lithium nitrate was selected as the electrolyte. The cathodes possess an areal density of ~2.0 mg cm^−2^. The Nyquist plot curves of SPCS-S and SPCT-S electrodes were shown in Supplementary Information Figure S8. It is worth noting that SPCS-S exhibits 21% lower charge transfer resistance (R_ct_) than SPCT-S, suggesting a higher electrochemical performance of SPCS-S electrode. Figure 5a shows the galvanostatic charge/discharge curves of the initial five cycles of SPCS-S hybrid at 0.2 C (1 C = 1675 mA g^−1^). The cyclic voltammogram (CV) curves of SPCS-S cell were carried out after 100 charge/discharge tests at 0.2 C, as shown in Fig. 5b, at a scan rate of 0.2 mV s^−1^ within the voltage range of 1.8 V to 2.8 V. The corresponding charge/discharge and CV curves of SPCT-S hybrid are shown in Supplementary Information Figure S9. Both SPCT-S and SPCS-S cathodes exhibit typical charge-discharge behaviour of sulfur cathode, which can be divided into two stages during discharge process: element sulfur (S_8_) to high-ordered polysulfides (S_7_^2−^, S_6_^2−^, S_4_^2−^) and further reduction to low-order polysulfides (S_2_^2−^, S^2−^), corresponding to the two reduction peaks in CV curves^1^,^6^,^7^.

The first stage corresponds to the upper-plateau at 2.15-2.4 V in the charge/discharge curves and the reduction peak at ~2.35 V in CV curves^15^. In the second stage, as Li_2_S_2_ and Li_2_S are formed, and the corresponding voltage is lowered, a long lower-plateau and a larger reduction peak can be observed. The charge curves also display two potential plateaus: a linear sloping (corresponding to the oxidation peak at ~2.3 V in CV curves) and a short plateau at relatively high voltage ranges (corresponding to the oxidation peak at ~2.4 V in CV curves), corresponding to the transformation of L_2_S_2_ or Li_2_S to low-order lithium sulfides and further convert to high-order lithium sulfides or elemental sulfur, respectively^42^. The two plateaus (the two oxidation peaks) were unconspicuous, owing to the high overvoltage when Li_2_S_2_ or Li_2_S converts to lithium ploysulfides. The SPCS and SPCT cathode respectively delivered a discharge specific capacity of ~1099.2, 954.6 mAh g^−1^ in the first cycle at 0.2 C, with the initial Coulombic efficiency of 95.8% and 92.2%. The low Coulombic efficiency was attributed to the solid electrolyte interface (SEI) and the irreversible reaction of sulfur, caused by the insoluble and irreversible Li_2_S_2_ (or Li_2_S) depositing at the interface between electrode and electrolyte.

**Figure 6:**
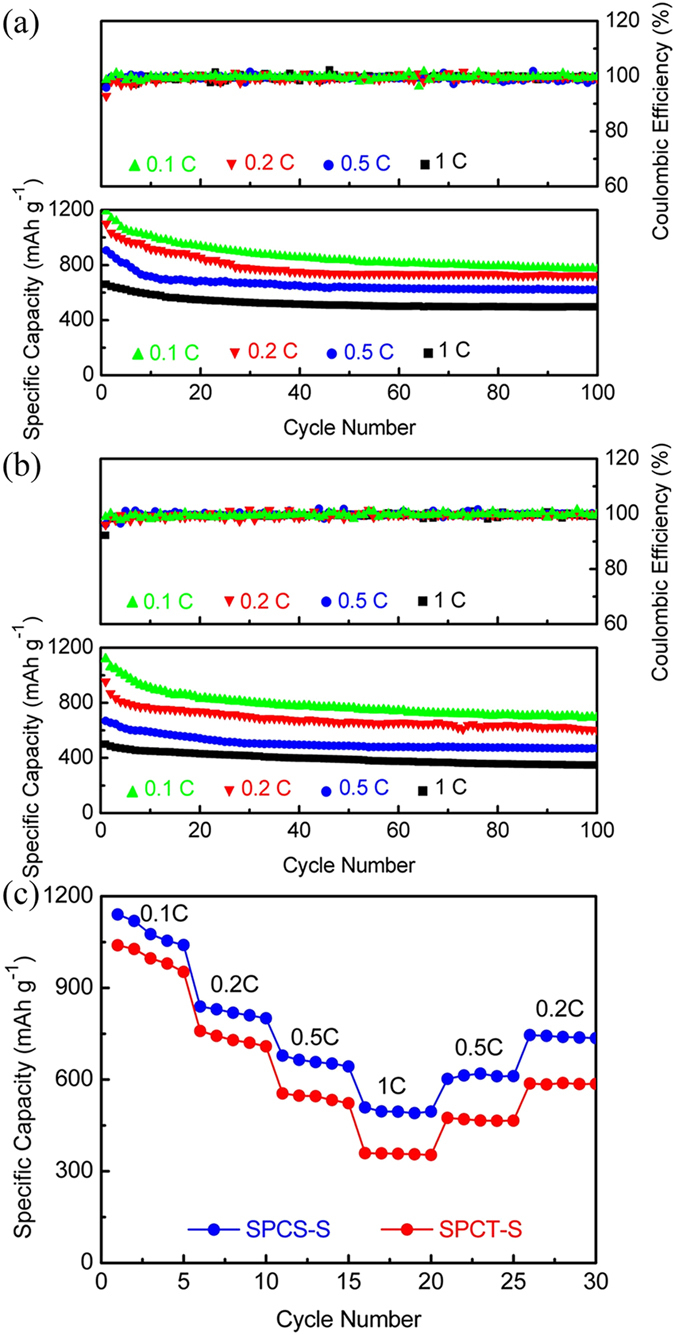
The cycling performance and Columbic efficiency of (**a**) SPCS-S and (**b**) SPCT-S and (**c**) the rate capability of SPCS-S and SPCT-S hybrids.

The cycling performance and Columbic efficiency of SPCS-S (Fig. 6a) and SPCT-S (Fig. 6b) cathodes with different current density was investigated. The SPCS-S cathode can achieve the initial specific capacity of 1189.5, 1099.2, 906.5 and 660.3 mAh g^−1^ at rates of 0.1 C, 0.2 C, 0.5 C, and 1 C, respectively. After 100 charge/discharge tests, the cathode displays a stable capacity of 778.9, 722.5, 619.8, and 476.4 mAh g^−1^ at the same rates from 0.1 C to 1 C, with the corresponding capacity retentions of 65.0, 65.7, 68.4 and 72.1%, respectively. While for SPCT, the cathode shows the initial capacity of 1118.6, 954.6, 668.4, and 499.0 mAh g^−1^ at 0.1 C, 0.2 C, 0.5 C and 1 C, respectively, which can maintain at 693.4, 601.6, 450.3, and 348.1 mAh g^−1^ after 100 cycles, corresponding the capacity retention of 62.0, 63.0, 67.4 and 69.6%. For both SPCS-S and SPCT-S cathodes, the average Coulombic efficiency with different rates is obtained around the level of 99.5% during the 100 cycles. The rate capability of SPCS-S and SPCT-S hybrids was evaluated by galvanostatic method at various current rates, as shown in Fig. 6c. The capacity recuperability of SPCS-S hybrid can achieve ~90%, higher than that of SPCT-S hybrid (~81%) when the current returned to 0.2 C.

**Figure 7:**
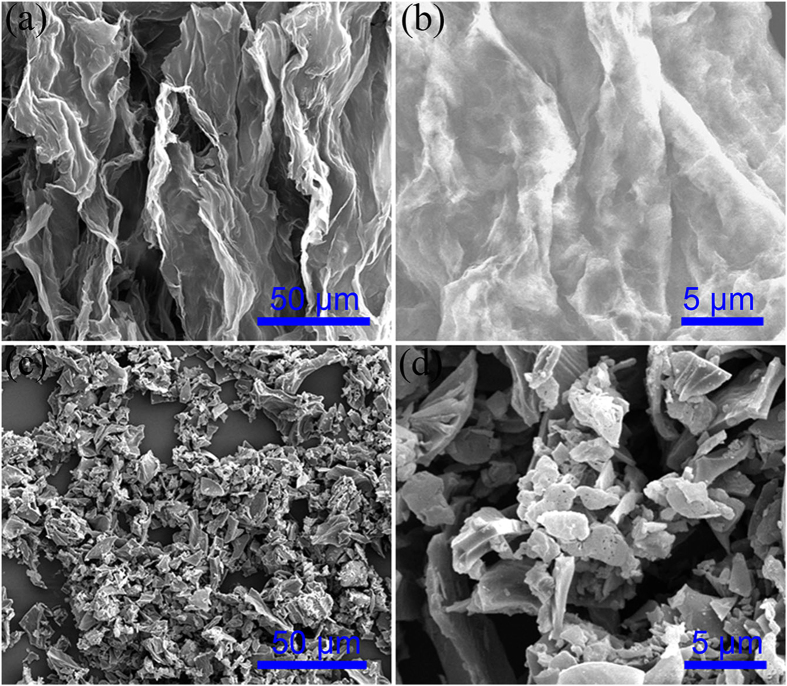
Morphology characterization of active materials after 50^th^ discharge tests at 0.2C (**a**,**b**) SPCS-S; (**c**,**d**) SPCT-S.

To demonstrate the superiorities of carbon sheets of SPCS, we investigated the morphology of SPCT-S and SPCS-S hybrids after 50th discharge tests at 0.2 C (Fig. 7). The coin cells were disassembled in the argon-filled glovebox, and the cathodes were firstly washed several times with dimethyl carbonate (DMC) to remove the soluble species, followed by washing and centrifuging several times with tetrahydrofuran (THF), and finally dried at room temperature in argon-filled glovebox to collect active materials. Figure 7a,b exhibited the low and high magnification SEM images of SPCS-S after 50^th^ discharge tests at 0.2 C. It is clearly observed that a significant structural preservation of SPCS-S occurred after repeatedly charge/discharge tests, demonstrating the superior flexibility of carbon sheets in SPCS. This character endowed the SPCS-S cathodes with well accommodating of the volume changes and the structural retention, leading to its excellent cyclic stability. Figure 7c,d displayed the SEM images of SPCT-S after 50^th^ discharge tests at 0.2 C, and the tube structure of SPCT was destroyed after repeatedly discharge/charge tests due to the volume expansion/shrink of sulfur particles, revealing its poor stability during cycling. The elemental mapping of SPCS-S and SPCT-S after 50^th^ discharge tests at 0.2 C was shown in Supplementary Information Figure S10 and Figure S11, and the appearance of oxygen was mainly from the oxidation of sulfur species during the SEM sample preparing process. It is worthwhile to note that a large amount of sulfur species were detected on the surface of carbon sheets in SPCS-S (Supplementary Information Figure S10), suggesting the high utilization of active material and cyclic stability of SPCS-S cathode.

The more excellent capacity, rate performance and cycling stability arise for SPCS from three effects: (i) the N-doping of SPCS enhancing the reduction of sulfur; (ii) the larger specific surface area being applied to absorb/immobilize sulfur species; (iii) the flexibility of carbon sheets in SPCS endowing cathode with well accommodating of the volume changes and a structural preservation; (iv) the uniformly distribution of sulfur enlarging the interface between conductive substrate (SPCS) and sulfur species and finally resulting in high rate performance.

## Conclusion

In summary, the SPCS obtained from organic waste shaddock peel through hydrothermal treatment and carbonization is a promising, low-cost, truly green and renewable carbon material that serves as hosts for sulfur cathode with superb electrochemical performance. The SPCS possesses the 3D interconnected porous network for electronic delivery, electrolyte channel and sulfur species absorption/trap. The large specific surface area of SPCS and uniform sulfur-distribution in SPCS-S enlarge the interface between conductive substrate (SPCS) and sulfur species. The flexibility of carbon sheets in SPCS endows cathode with well accommodating of the volume changes and a structural preservation. As a result, the SPCS-S cathode exhibits an excellent cycling stability and rate property. Meanwhile, the SPCT was generated by direct carbonization, as a contrast with SPCS for sulfur cathode. The results demonstrated that the carbon materials obtained from shaddock peel or other organic wastes are economically and commercially encouraging for the low-cost, recyclable, and green energy storage systems.

## Methods

### Synthesis of SPCT and SPCS

SP was cleaned using deionized (DI) water with several times and freeze-dried for 24 h. The dried SP was carbonized in quartz tube furnace at 900 °C for 2 h with a heating rate of 10 °C min^−1^ under argon (Ar) and hydrogen (H_2_) atmosphere (Ar and H_2_ flow rate: 400 mL min^−1^ and 40 mL min^−1^). The samples were washed with boiled 1 M HCl and abundant DI water, and then SPCT was obtained by drying in vacuum oven at 80 °C for 24 h.

Shaddock peel was chopped into blocks with a mass of 0.5 g, and then cleaned using DI water for several times. Thereafter, the cleaned SP and 8 mL KOH (1 M) aqueous solution were sealed in a 15-mL Teflon-lined stainless steel autoclave and maintained at 140 °C for 12 h. After self-cooled to room temperature, a gray SP hydrogel can be obtained by being soaked in DI water for several days to residual impurity ions, and then SP aerogel was freeze-dried for 24 h. For SPCS preparation, the as-prepared SP aerogel was carbonized by the same method of SPCT. The SPCS were obtained after washing and drying by the same method of SPCT.

### Synthesis of SPCT-S and SPCS-S hybrids

The SPCT-S and SPCS-S hybrids were prepared by a melting diffusion method. 0.7 g sublimed sulfur and 0.3 g as-prepared samples (SPCS or SPCT) were mixed by grind milling for 30 min in an agate mortar. Then, the mixture was placed inside quartz tubes that were sealed under vacuum, and were transfer to a quartz tube furnace under 155 °C for 10 h with a heating rate of 5 °C min^−1^. After cooled down to room temperature, the SPCT-S and SPCS-S hybrids were obtained.

### Materials characterization

The X-ray diffraction (XRD) was measured by a D/max-2000 diffractometer with Cu-K*α* irradiation (*λ* = 1.54056 Å) in the 2θ range from 10 to 80 degrees. Raman spectra were carried out on Bruker Optics Senterra R200-L Raman scattering microscope with a wavelength of 633 nm. The electronic conductivity of samples was measured by Hall Effect Measurement System (Swin HALL 8800, Chinese Taipei) at room temperature. Nitrogen adsorption-desorption isotherms were measured on a Micromeritics TriStar II 3020 at 77 K, and the surface areas were calculated using the Brunauer-Emmett-Teller (BET) method. The morphology was characterized by field scanning electron microscope (FESEM, JSM-6700) and transmission electron microscope (TEM, H-7650). The surface elemental composition of samples was analysed by X-ray photoelectron spectroscopy (XPS, ESCALAB Mark II, VG) with Al K*α* X-ray source. The sulfur content was determined by thermogravimetric analysis (TGA, ZRY-2P).

### Electrochemical characterization

The SPCT-S and SPCS-S cathodes were prepared via a slurry coating method. The slurry constituted of 90 wt% the as-prepared active material (SPCT-S or SPCS-S) and 10 wt% polyvinylidene fluoride (PVDF), N-methyl pyrrolidone (NMP) was used as solvent. The cathodes with the diameter of 10 mm and the loading of active materials of ~2.0 mg cm^−2^ were obtained through the slurry coated on aluminium foil current collect and dried at 60 °C in vacuum for 24 h. The preparation of SPCS, SPCT and carbon black (purchased from SHENZHEN KEJINGSTAR TECHNOLOGY LTD.) electrodes was by the same method, containing 90 wt% SPCS (or SPCT, carbon black) and 10 wt% PVDF. CR2032-type coin cells were assembled in an argon-filled glove box with lithium foil as the anode. The separator was purchased from Cellgard (model 2400). The electrolyte was 1.0 M lithium bis(trifluoromethane) sulfonamide dissolved in the mixture of 1,3-dioxolane and 1,2-dimethoxymethane (volume ration 1:1) and 0.1 M lithium nitrate as an electrolyte additive. The electrochemical properties of SPCT-S and SPCS-S cathodes were measured by galvanostatic measurement in a potential range of 1.8–2.8 V (*vs.* Li^+^/Li) by using a LAND CT2001A battery test system at room temperature. Cyclic voltammetry measurements were tested in the same potential range at a scanning rate of 0.1 mV s^−1^ under an electrochemical workstation (PARSTAT 4000, Princeton, USA.) Electrochemical impedance spectrum (EIS) measurements were performed using an electrochemical workstation (PARSTAT 4000, Princeton, USA.) within a frequency range of 100 kHz to 0.01 Hz and a potentiostatic signal amplitude of 10 mV.

## Additional Information

**How to cite this article**: Lu, S. *et al.* A Sheet-like Carbon Matrix Hosted Sulfur as Cathode for High-performance Lithium-Sulfur Batteries. *Sci. Rep.* **6**, 20445; doi: 10.1038/srep20445 (2016).

## Supplementary Material

Supplementary Information

## Figures and Tables

**Table 1:** Surface elemental composition, textural properties, carbon structure and electronic conductivity of SP, SPCT and SPCS.

Sample	Surface chemical analysis (XPS)				Textural properties		Carbon Structure		Conductivity (S cm^−1^)
	C (wt%)	O (wt%)	N (wt%)	K (wt%)	*S*_*BET*_ (m^2^ g^−1^)	*V*_*t*_^*b*^ (cm^3^ g^−1^)	*d*_002_ (Å)	*I*_G_/*I*_D_^*c*^	
SP	48.09	45.1	1.80	1.01	51.1	0.04	–	–	–
SP^*d*^	44.19	48.52	1.76	4.05	149.6	0.13	–	–	–
SPCT	91.15	7.08	1.77	–	484.3	0.40	3.78	1.02	–
SPCS	90.06	8.25	1.69	–	937.1	0.82	4.19	0.44	107.2

^*a*^Specific surface area. ^*b*^Total pore volume. ^*c*^the intengrated intensities of G-band and D-band. ^*d*^SP with hydrothermal treatment.
