# Supplementary material for: A Sheet-like Carbon Matrix Hosted Sulfur as Cathode for High-performance Lithium-Sulfur Batteries
Source: Sci Rep. 2016 Feb 4;6:20445. doi: 10.1038/srep20445 (PMC4740814; doi:10.1038/srep20445)
Supplement: Supplementary Information [file srep20445-s1.pdf]

Supplementary Information

A Sheet-like Carbon Matrix Hosted Sulfur as Cathode for High-performance Lithium-Sulfur Batteries

Songtao Lu[1,+], Yan Chen[1,+], Jia Zhou[1], Zhida Wang[1], Xiaohong Wu[1,*], Jian Gu[2], Xiaoping Zhang[2], Aimin Pang[2], Zilong Jiao[3] and Lixiang Jiang[3]

[1]Department of Chemistry, Harbin Institute of Technology, Harbin, Heilongjiang 150001, PR China

[2] Hubei Institute of Aerospace Chemotechnology, Hubei 441003, PR China

[3]Science and Technology on Reliability and Environmental Engineering Laboratory, Beijing Institute of Satellite Environment Engineering, Beijing 100094, PR China

*Correspondence and requests for materials should be addressed to X.H.W. (email: wuxiaohong@hit.edu.cn)

[+]These authors contributed equally to this work

Table and Figures

Table S1 The elemental analysis results from EDS with SEM.

Sample	Surface elemental composition (EDS)			
	C (wt%)	O (wt%)	N (wt%)	K (wt%)
SP	47.54	50.86	-	1.6
SP[a]	43.93	51.91	-	4.16
SPCT	89.64	10.36	-	-
SPCS	87.82	12.18	-	-

[a]SP with hydrothermal treatment.

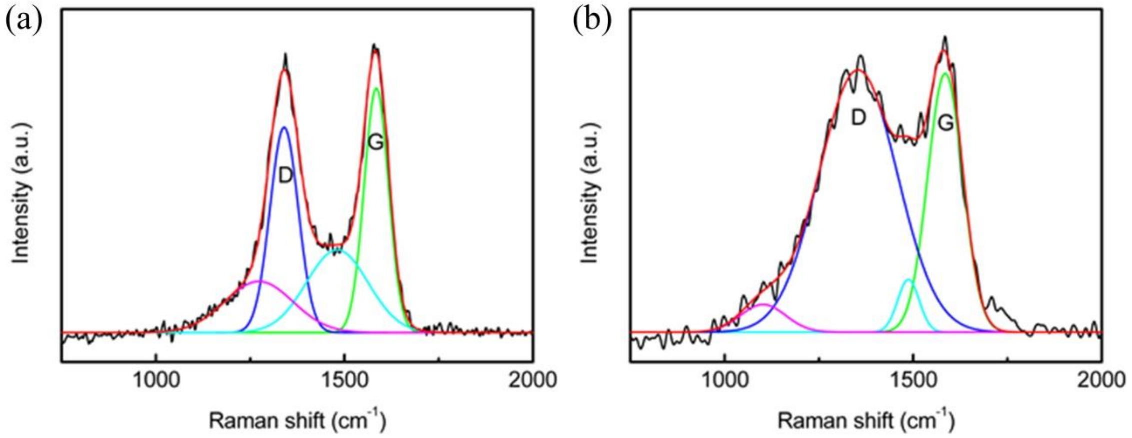

Figure S1 Fitted Raman spectra of (a) SPCT and (b) SPCS.

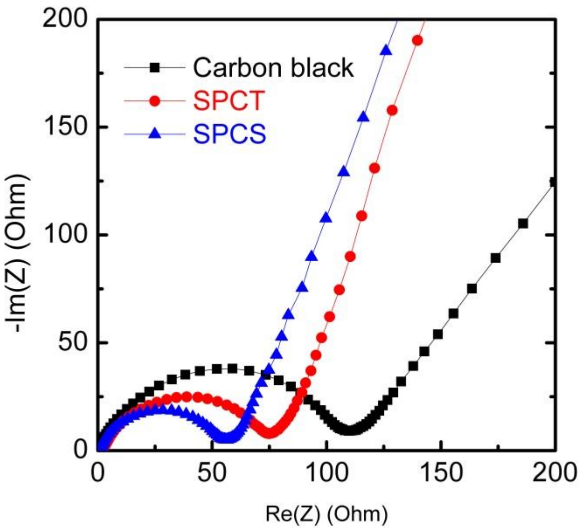

Figure S2 Nyquist plot curves of electrodes prepared from SPCS, SPCT and carbon black.

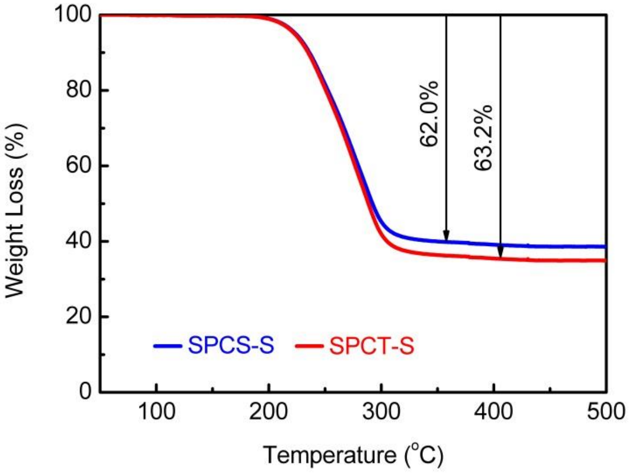

Figure S3 The thermogravimetric analysis of SPCT-S and SPCS-S hybrids.

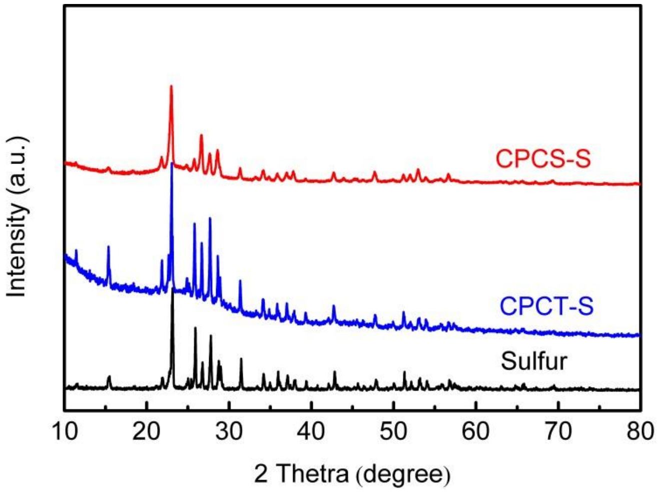

Figure S4 The XRD patterns of SPCT-S and SPCS-S hybrids.

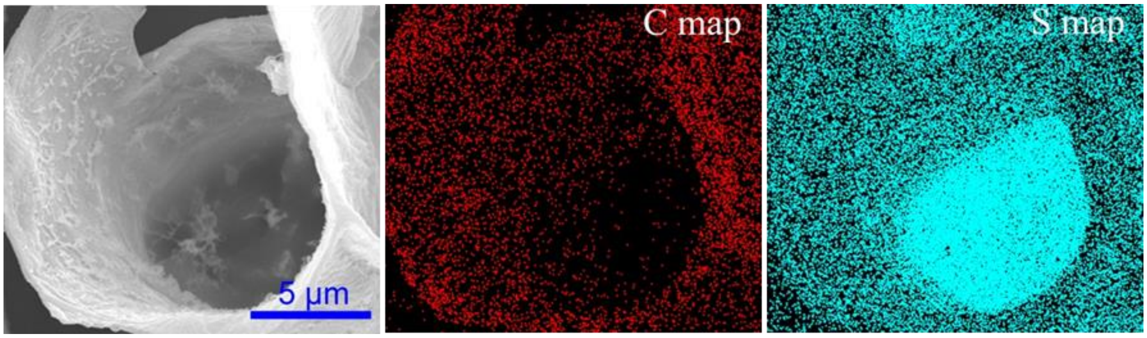

Figure S5 The SEM images and mappings of SPCT-S hybrid.

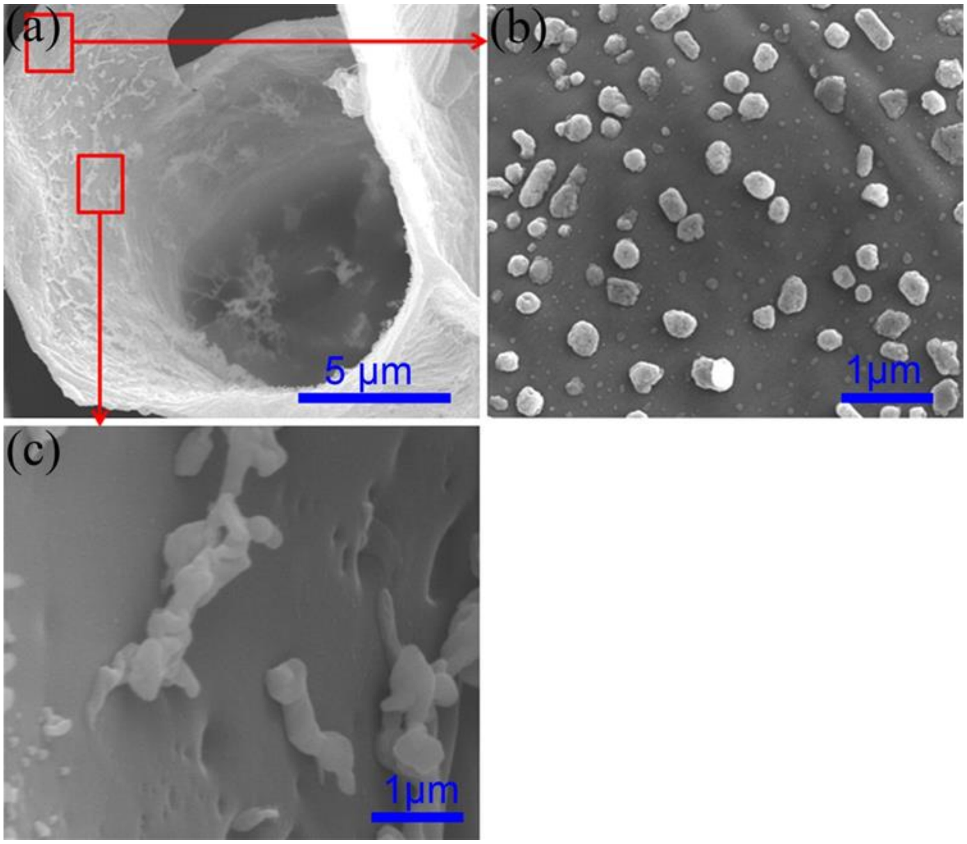

Figure S6 The higher magnification SEM images of SPCT-S hybrid from different regions.

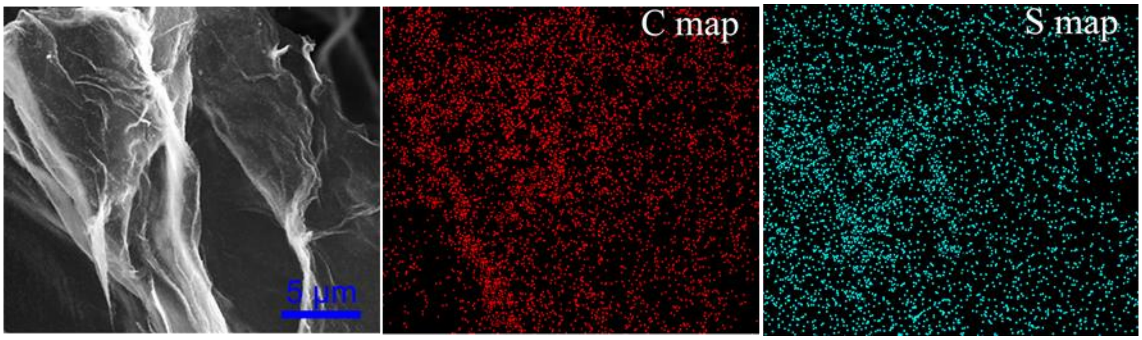

Figure S7 The SEM image and elemental mappings of SPCT-S hybrid.

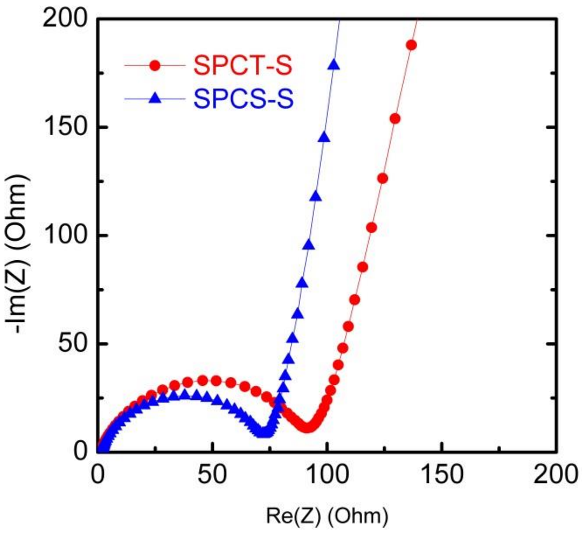

Figure S8 Nyquist plot curves of electrodes prepared from SPCS-S and SPCT -S.

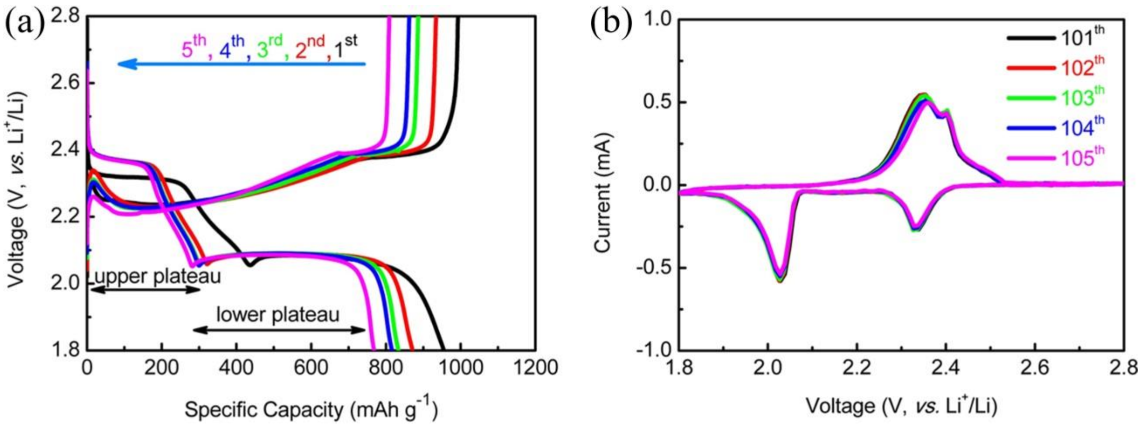

Figure S9 Electrochemical performance of SPCT-S hybrid, showing the behaviors of (a) the galvanostatic charge/discharge curves of SPCT-S hybrid at 0.2 C and (b) the cyclic voltammogram curves of SPCT-S hybrid after 100 charge/discharge tests at 0.2 C.

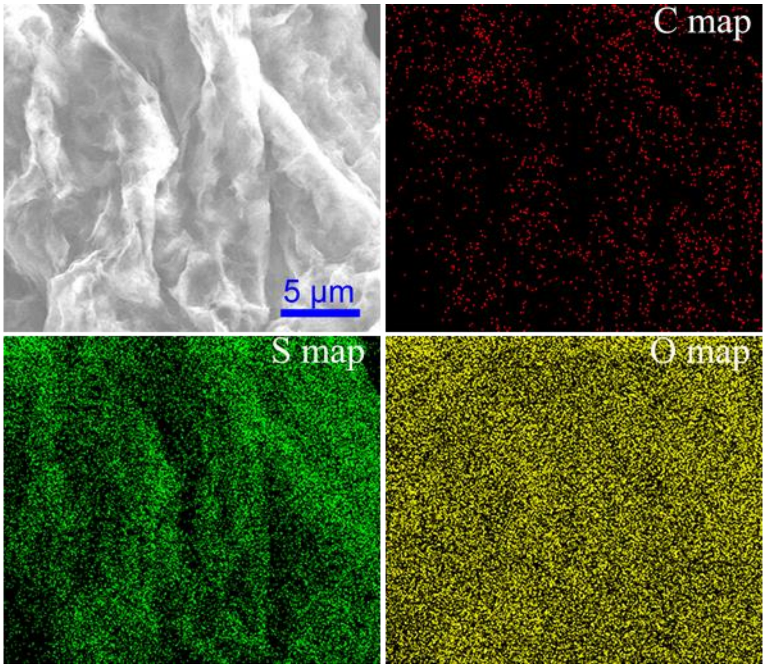

Figure S10 The SEM image and elemental mappings of SPCS-S hybrid after 50th discharge tests at 0.2 C.

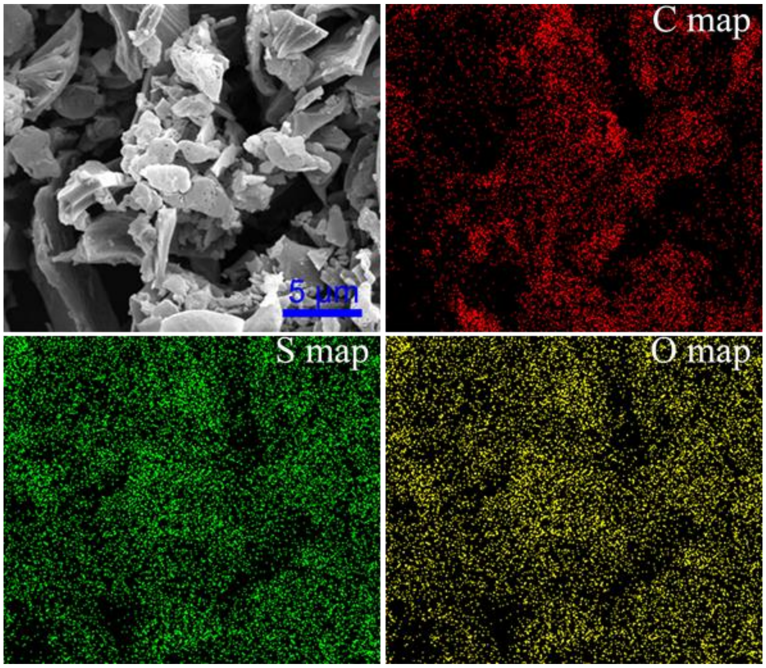

Figure S11 The SEM image and elemental mappings of SPCS-S hybrid after 50th discharge tests at 0.2 C.
